# Supplementary material for: Targeted inhibition of activated protein C by a non-active-site inhibitory antibody to treat hemophilia
Source: Nat Commun. 2020 Jun 12;11:2992. doi: 10.1038/s41467-020-16720-9 (PMC7293249; doi:10.1038/s41467-020-16720-9)
Supplement: Supplementary file 1 — Supplementary Information [file 41467_2020_16720_MOESM1_ESM.pdf]

SUPPLEMENTARY INFORMATION

Targeted Inhibition of Activated Protein C by a Non-Active-Site Inhibitory Antibody to Treat Hemophilia

Zhao et al.

RESTRICTED

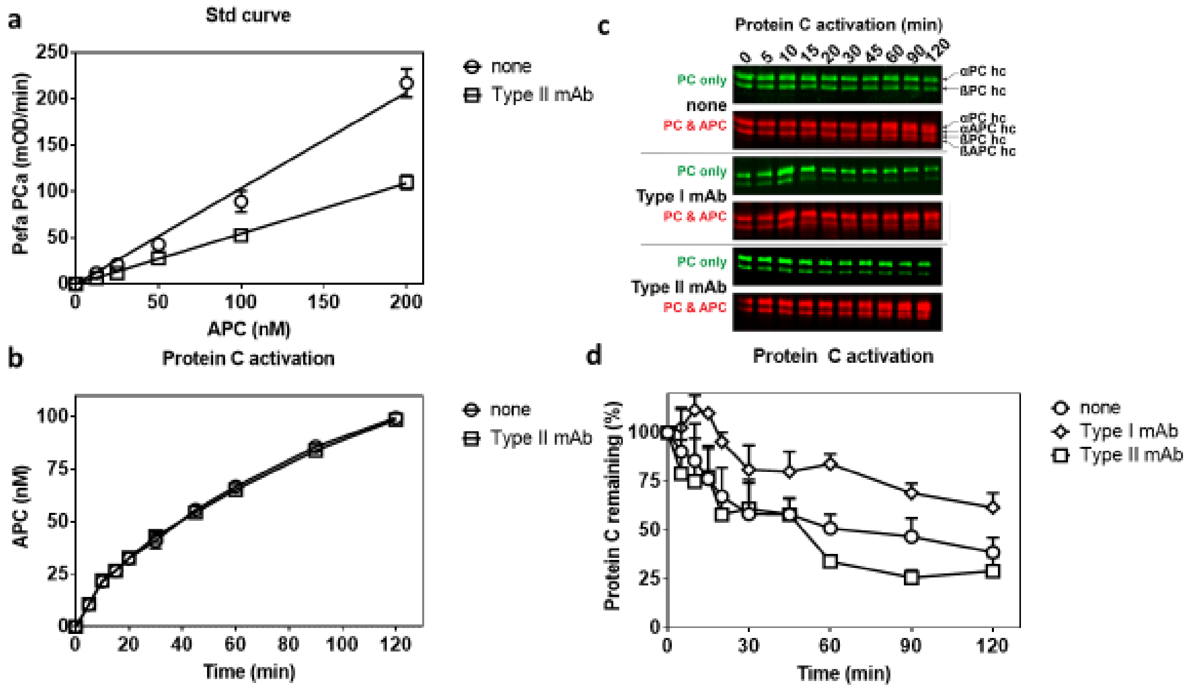

Supplementary Figure 1. Effects of type I and type II mAbs on protein C activation. The effect of the type II mAb on protein C activation was determined using a chromogenic assay for APC generation. (**a**) To determine the effect of the type II mAb on protein C activation individual standard curves for the cleavage of Pefachrome PCa by APC in the presence and absence of the type II mAb (1 µM) were generated. (**b**) Protein C (100 nM) was activated by thrombin (20 nM) and thrombomodulin (10 nM) in Hepes buffered saline containing 5 mM $CaCl_2$ and 0.1% BSA for up to 2 hours in the presence and absence of the type II mAb (1 µM). APC generation was determined by amidolytic activity after addition of hirudin (30 U/ml) and Pefachrome PCa (0.4 mM) and converted to nM APC using the appropriate standard curve (**a**). The effect of the type I mAb on protein C activation was determined by qualitative Western blot because the type I mAb inhibits the amidolytic activity of APC. (**c**) Western blot of protein C activation samples (100 nM PC, 5 nM thrombin, 10 nM thrombomodulin, 1 µM mAb) using a biotinylated protein C specific antibody (HPC4) detecting the heavy chain (hc) of protein C

RESTRICTED

(detected with streptavidin-IRdye800; green channel) and a polyclonal anti-protein C antibody (goat anti-PC; ERL) recognizing the heavy chain of both protein C and APC (detected with donkey anti-goat-IRdye680; red channel). The αPC-hc and βPC-hc bands reflect partial glycosylation at Asn329. (**d**) Quantification of protein C activation in the presence of no mAb, type I mAb, or type II mAb based on the disappearance of the αPC-hc and βPC-hc in the green channel and normalized to the corresponding lane signal between the αPC-hc and βAPC-hc bands in the red channel. Each point represents the average of 3 independent activation reactions analyzed on individual gels.

RESTRICTED

Supplementary Figure 2

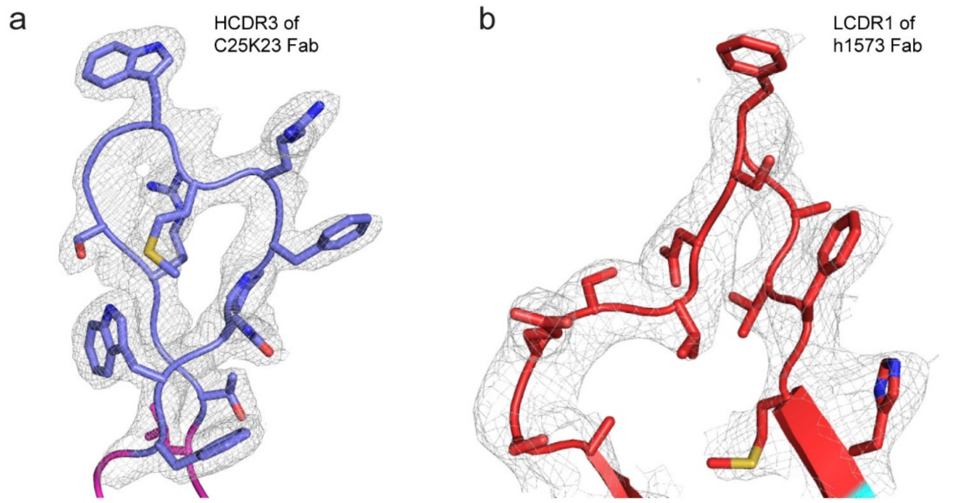

c

C25K23 (type I)	h1573 (type II)
H57	
D60	
E60-A	
S95	
K96	
S97	
T98	
T99	
	Y143
	S145
	S146
	R147
	E148
	K149
	E149-A
	A149-B
S169	
E170	
V171	
M172	
S173	S173
N174	
M175	
	G186
	R187
D189	
A190	
C191	C191
E192	E192
S195	
V213	
S214	
W215	
G216	G216
E217	E217
G219	G219
C220	C220
	G221
L221-A	L221-A
	L222
	H223
	N224

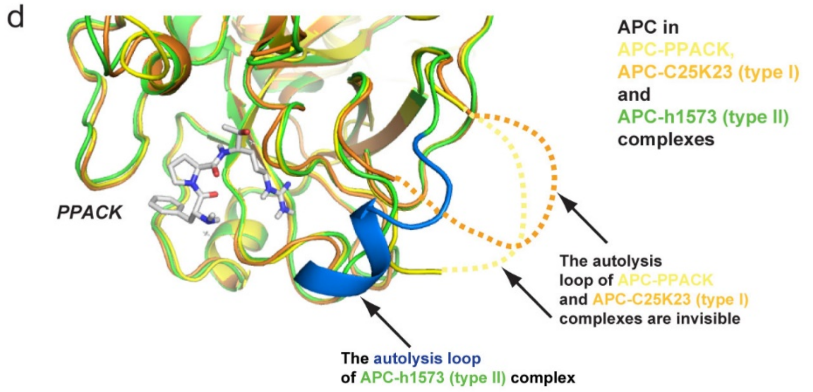

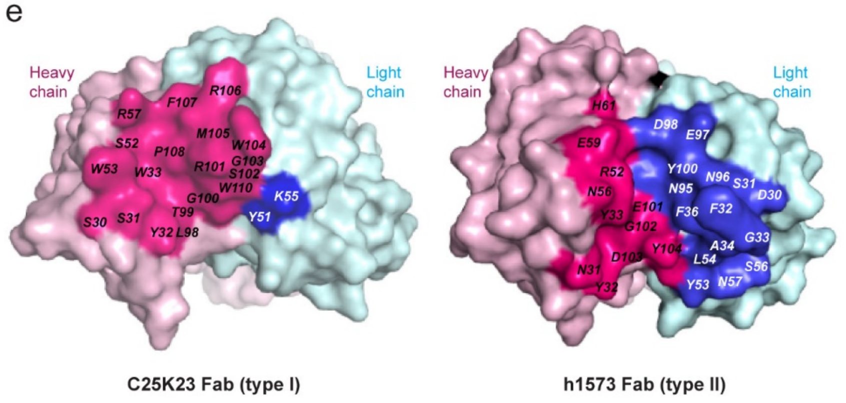

RESTRICTED

Supplementary Figure 2. Electron density map, epitope and the autolysis loop of APC, and paratopes of the C25K23 (type I) Fab and h1573 (type II) Fab. (a) 2Fo-Fc electron density map contoured at 1.0 σ of the HCDR3 loop of C25K23 Fab. **(b)** 2Fo-Fc electron density map contoured at 1.0 σ of the LCDR1 loop of h1573 Fab.**(c)** APC residues as binding epitopes of the C25K23 (type I) and h1573 (type II) Fabs. Active site residues of APC are marked cyan. **(d)** Superimposition of APCs in APC-PPACK (PDB code: 1AUT) (yellow), APC-C25K23 Fab (orange), and APC-h1573 Fab (green) complex structures. The autolysis loop is shown as marine in the APC-h1573 complex structure, and dash lines in the other two structures where it was not defined. (**e)** Paratopes of the C25K23 (type I) and h1573 (type II) Fabs. For C25K23 Fab, the paratope (within 5 Å to APC) comprises residues S30-W33, S52, W53, R57, L98-P108, and W110 of the Fab heavy chain and residues Y51 and K55 of the Fab light chain. For h1573 Fab, the paratope (within 5 Å to APC) comprises residues D30-A34, F36, Y53, L54, S56, N57, N95-D98, and Y100 of the Fab light chain and residues N31-Y33, R52, N56, E59, H61, and E101-Y104 of the Fab heavy chain.

RESTRICTED

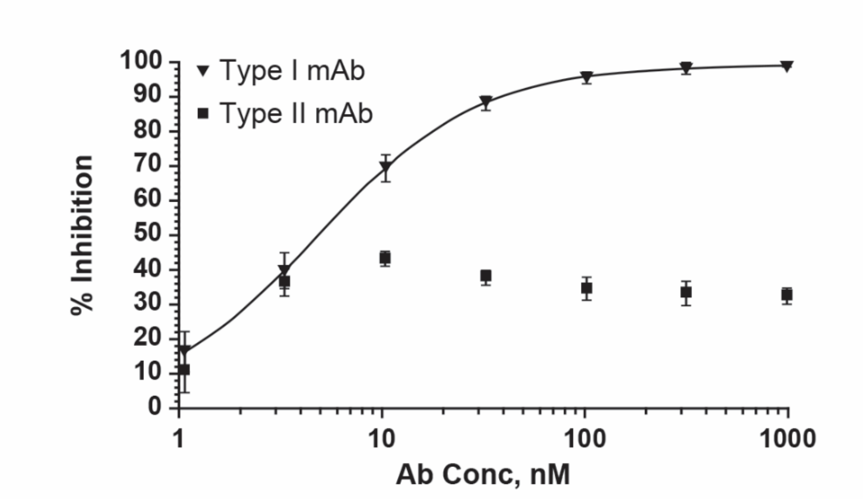

Supplementary Figure 3. Inhibition of APC's amidolytic activity by type I and type II mAbs. Raw data of APC amidolytic activity assays presented in Figure 3a are converted to % inhibition here. Type I mAb gave a maximal 98% inhibition of APC amidolytic activity with IC50 of 4.8 nM. In contrast, type II mAb showed a plateau of 43% inhibition of APC amidolytic activity at 10 nM.

RESTRICTED

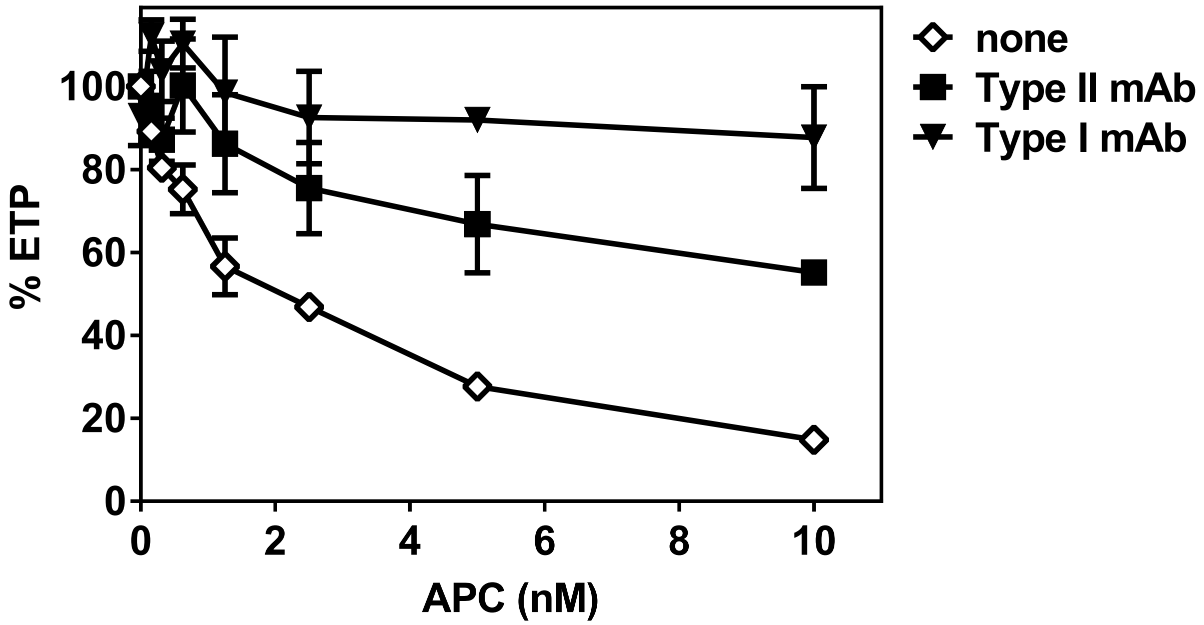

Supplementary Figure 4. Effect of type I and type II mAbs on thrombin generation in human HemA plasma. Thrombin generation was initiated in human hemophilia A plasma (George King Bio-Medical, Inc.) by 3 pM tissue factor (Innovin) in the presence of APC and type I or type II mAbs (100 nM). Thrombin generation was expressed as ETP and normalized to that in the absence of APC which was set to 100%. Each point represents the mean ± SEM of 3 independent experiments. Inhibition of APC anticoagulant activity (as indicated by the recovered % ETP) in HemA plasma was greater by type I than by type II.

RESTRICTED

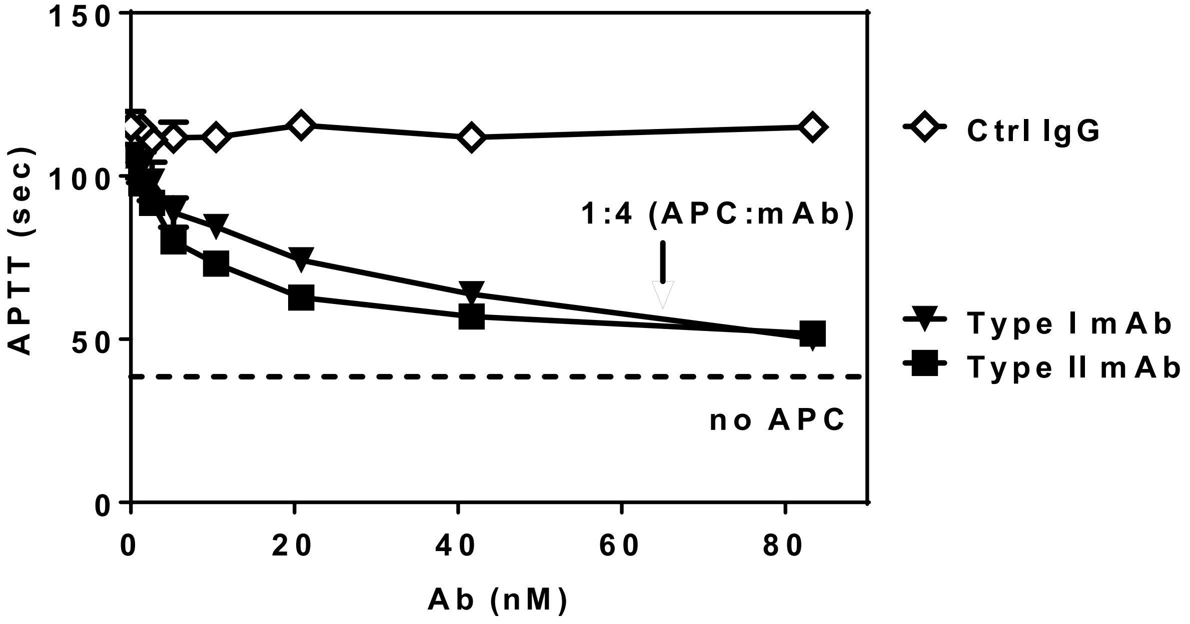

Supplementary Figure 5. Inhibition of APC anticoagulant activity by type I and type II mAbs. Anticoagulant activity of APC (16 nM) in normal pooled plasma was measured in an aPTT assay using kaolin and cephalin (C.K. Prest; Diagnostica Stago) in the presence of increasing concentrations of type I and type II mAb. The effect of type I and type II mAbs on APC barrier protective effects was measured in Figure 4d under conditions where the APC : mAb molar ratio was 1:4 and where >75% of APC's anticoagulant activity was inhibited.

RESTRICTED

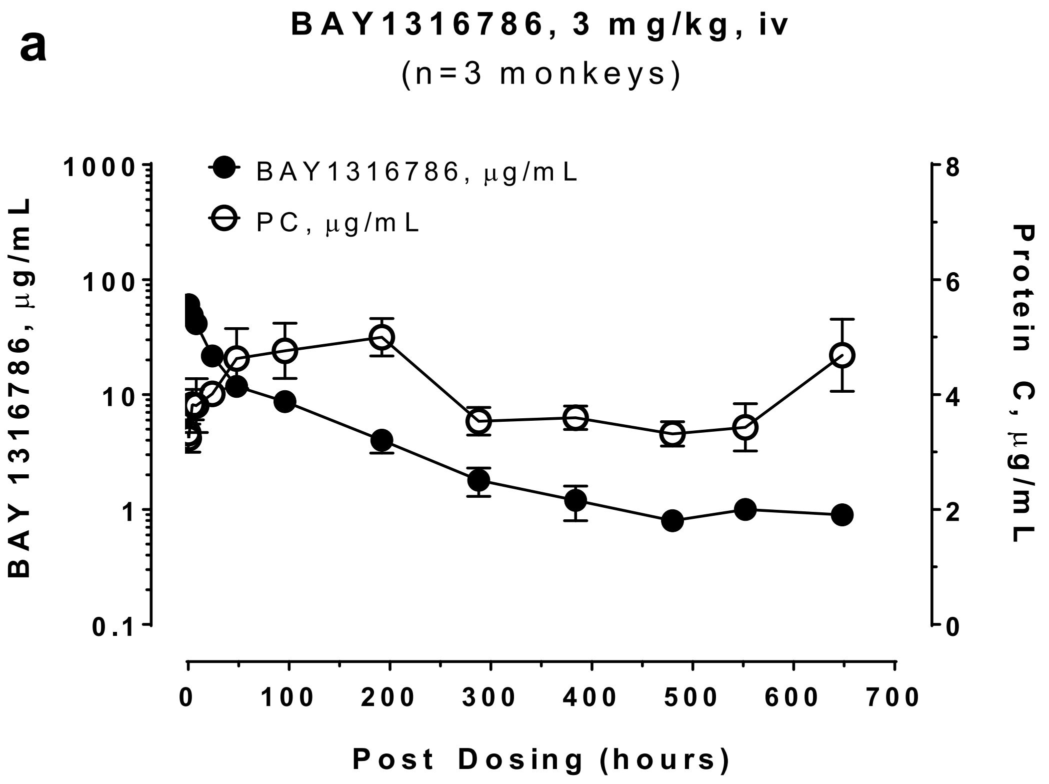
a
BAY1316786, 3 mg/kg, iv
(n=3 monkeys)
BAY1316786, µg/mL
PC, µg/mL
BAY 1316786, µg/mL
Protein C, µg/mL
1000
100
10
1
0.1
8
6
4
2
0
0
100
200
300
400
500
600
700
Post Dosing (hours)

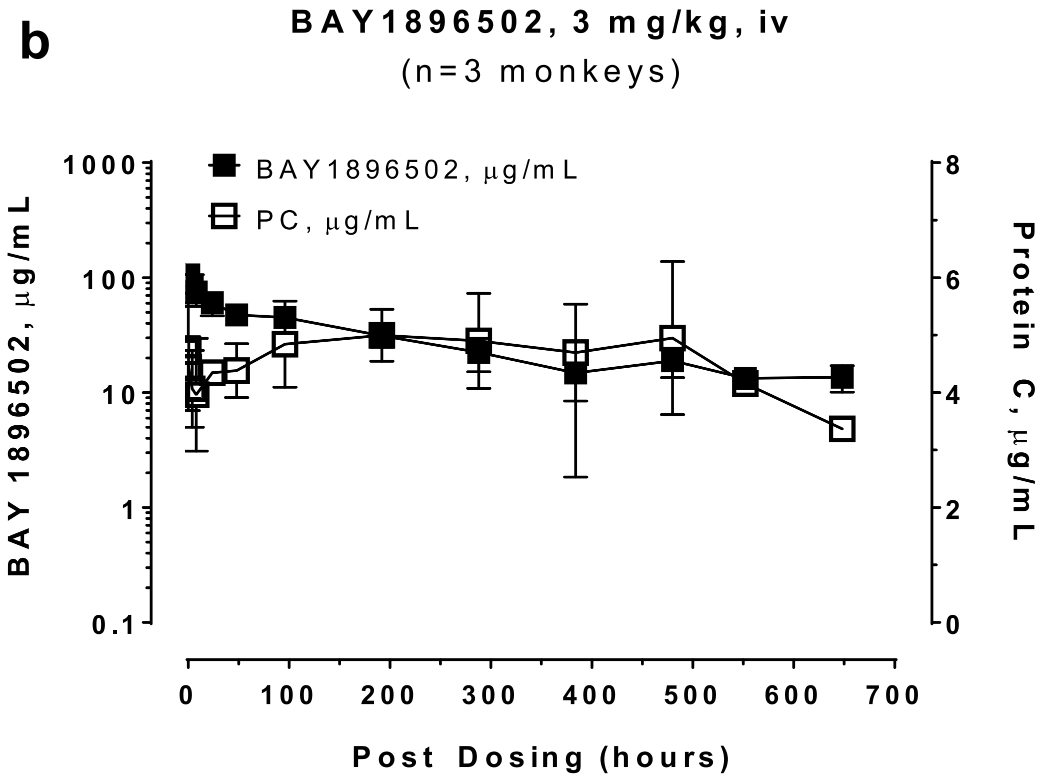
b
BAY1896502, 3 mg/kg, iv
(n=3 monkeys)
BAY1896502, µg/mL
PC, µg/mL
BAY 1896502, µg/mL
Protein C, µg/mL
1000
100
10
1
0.1
8
6
4
2
0
0
100
200
300
400
500
600
700
Post Dosing (hours)

RESTRICTED

Supplementary Figure 6. Plasma PC levels and drug levels in Cynomolgus monkeys dosed with type I and type II mAbs at 3 mg/kg, i.v. Plasma protein C (PC) concentration over the time in PK samples from Cynomolgus monkeys dosed with (**a**) type I anti-APC mAb (BAY1316786, closed circle), n=3 monkeys and (**b**) type II anti-APC mAb (BAY1896502, closed square), n=3 monkeys. Drug concentration were quantified with qualified ligand binding assays (LBA) using a goat-anti-human IgG (pre-absorbed by monkey IgGs) from SouthernBiotech (cat#2049-1) as captured antibody and HRP-goat-anti-human Fab as detection antibody followed by addition of OPD substrate. Significant improved PK profiles for type II mAb were observed over type I mAb (**Supplementary Table 2**). Plasma PC levels were measured using human Protein C ELISA kit (TSZ Scientific) and ranged from 3.0 to 6.47 μg/mL over the time. No significant changes in plasma PC levels were observed with either type mAb at the 3 mg/kg dose in these monkeys. Each point represents the mean ± SD of 3 monkey samples, and each data point are from triplicate measurements.

RESTRICTED

a

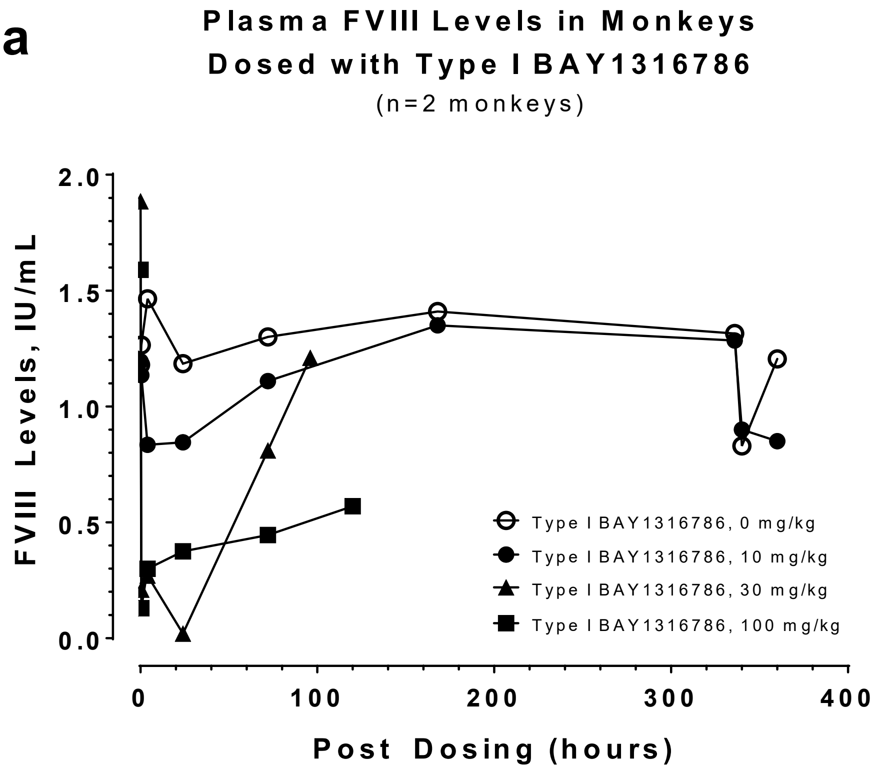

b

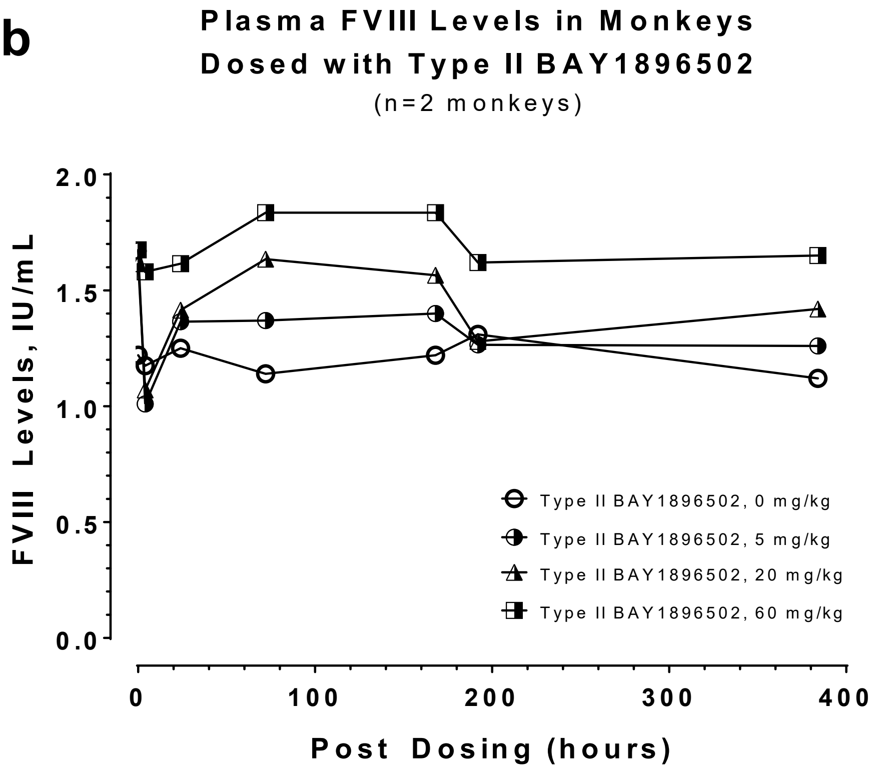

RESTRICTED

Supplementary Figure 7. Plasma FVIII activities in Cynomolgus monkeys dosed with anti-APC type I and type II mAbs. FVIII activities were measured over the time in plasma samples from Cynomolgus monkeys dosed with (**a**) type I mAb (BAY1316786 at 0, 10, 30, 100 mg/kg, i.v.), n=2 monkeys at each dose, and (**b**) type II mAb (BAY1896502 at 0, 5, 20, 60 mg/kg, i.v.), n=2 monkeys at each dose. FVIII activity was determined using the Chromogenix SP or SP4 Coatest kits (Diapharma, West Chester, OH). Human FVIII (12th BRITISH STANDARD FOR BLOOD COAGULATION FACTOR VIII CONCENTRATE, NIBSC Code 02/122) was used to generate the assay calibration curve. All samples were diluted 80-fold for the assay following the kit protocol and tested in duplicates. Administration of Type I mAb BAY1316786 at 10, 30 and 100 mg/kg caused a dose-dependent decrease in plasma FVIII activity in monkeys. At 10 mg/kg, Type I mAb triggered a mild decrease in FVIII activity at 4 and 24 hours, then FVIII returned to normal levels after 24 hours in both monkeys. However, at 30 and 100 mg/kg, Type I mAb produced a significant drop in FVIII activity starting at 30 min. One monkey in the 30 mg/kg treatment group died 45 minutes post dosing, and the remaining 3 monkeys in the 30 and 100 mg/kg groups required unscheduled euthanasia at various time points due to the severity of the adverse advents per protocol. All animals dosed with Type II BAY1896502 at 5, 20, and 60 mg/kg maintained steady state levels of FVIII over 16-day study period and no clinical signs of coagulation-mediated consumption of FVIII.

RESTRICTED

a **b**

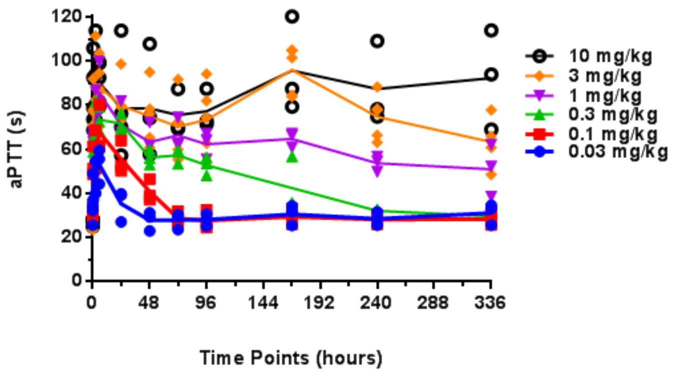

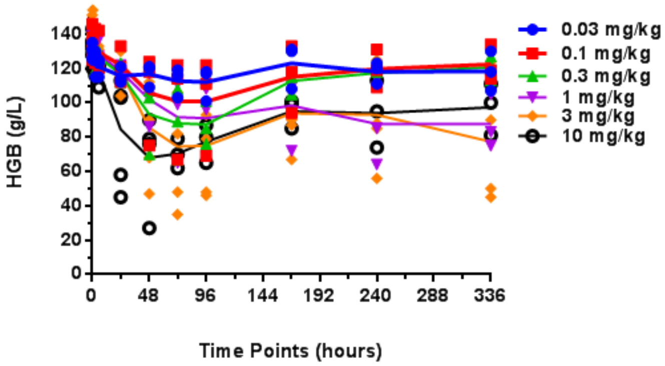

Supplementary Figure 8. Characterization of the acquired Hem A model in cynomolgus monkeys. (**a**) aPTT clotting time in seconds (s) and (**b**) hemoglobin (HGB) concentration in monkeys after administration of the anti-FVIII antibody BO2C11-cynoIgG1 at doses ranging between 0.03-10 mg/kg. Blood samples were collected at 0, 0.5, 2.5, 5.5, 24, 48, 72, 96, 168, 240, and 336h after dosing. A sustained aPTT prolongation was observed when monkeys were treated with 1, 3, 10 mg/kg of BO2C11-cynoIgG1. The prolong aPTT at 3 and 10 mg/kg BO2C11-cynoIgG1 was accompanied by a severe reduction of hemoglobin. Thus the 0.1 and 1 mg/kg BO2C11-cynoIgG1 dose were selected for establishing the intramuscular hemorrhagic anemia model.

RESTRICTED

a **b**

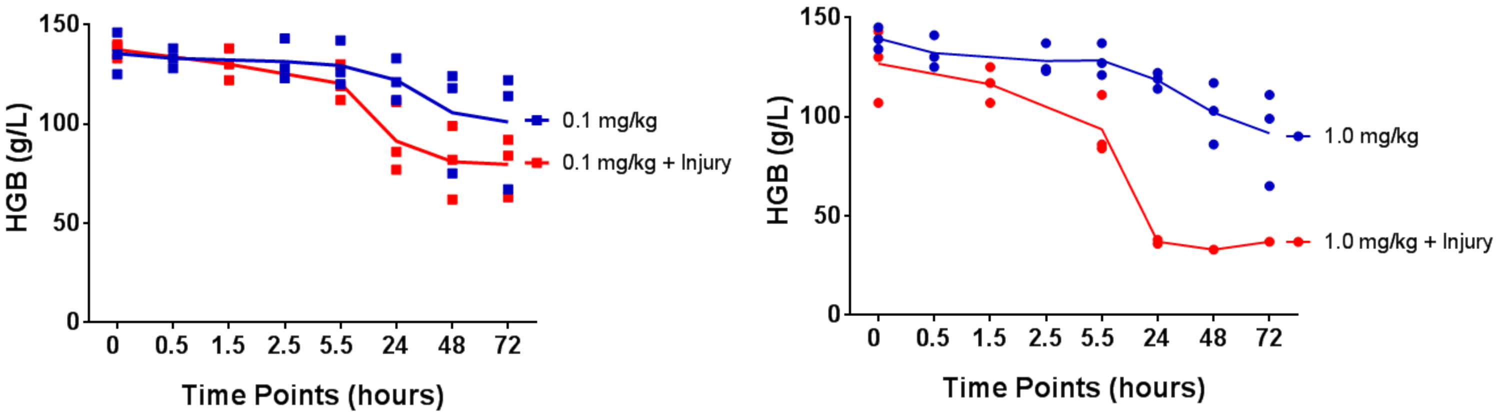

Supplementary Figure 9. Development of an intramuscular hemorrhagic anemia bleeding model in Hem A monkeys. Cynomolgus monkeys were treated with 0.1 mg/kg (**a**) or 1.0 mg/kg (**b**) of anti-FVIII antibody BO2C11-cynoIgG1 at t = 0. Levels of hemoglobin (HGB in g/L) in blood samples over time were measured using Sysmex XT 2000i Automated Hematology Analyzer (Systemex Corporation). Blood samples were collected at 0, 0.5, 1.5, 2.5, 5.5, 24, 48, and 72h. At t = 2 hours, bleeding was induced in the monkeys by inserting an 18-G needed 1-cm-deep into muscles at 16 sites (four sites in each thigh, two sites in each upper arm and two sites in each forearm). Anesthesia was maintained until the completion of this injury procedure. Anti-FVIII antibody led to a dose-dependent hemorrhagic anemia, and the needle injury further exacerbated the hemoglobin loss over time. A dose of 0.1 mg/kg anti-FVIII antibody BO2C11-cynoIgG1 was selected as the optimum dose to induce hemophilia for the intramuscular hemorrhagic anemia bleeding efficacy model. Efficacy data are shown in Figure 5j.

RESTRICTED

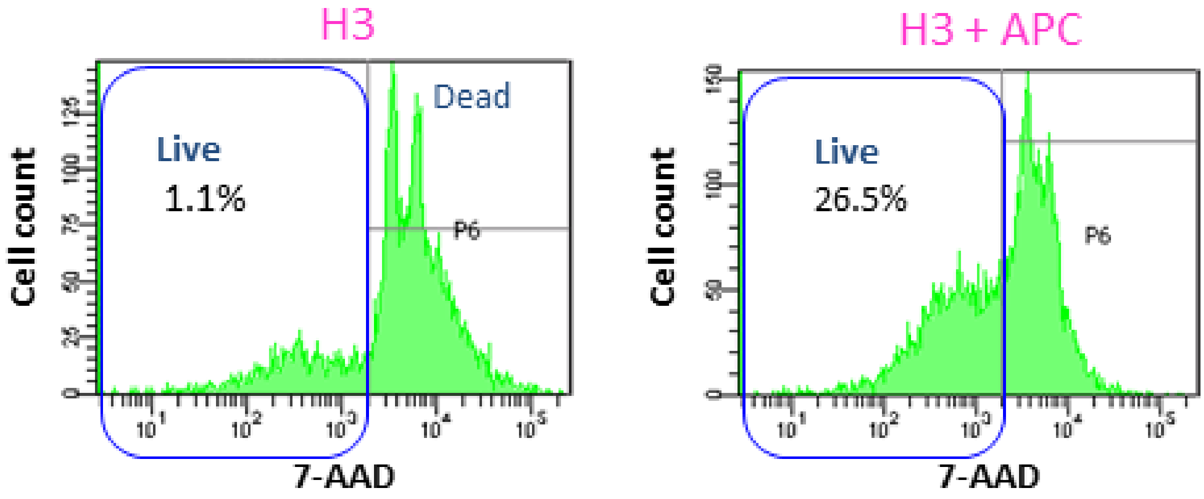

Supplementary Figure 10. Example of gating strategy used in Figure 4a. HUVEC cells were seeded at 2×10^6 cells per mL 1 day before the experiment. Confluent monolayers of HUVECs were incubated with 20 nM APC in the presence of mAb (0, 3, 30, 300 nM). Apoptosis was induced by incubation with calf thymus histone (Sigma-Aldrich) at 2 µM for 4 h. HUVECs were detached from the plate by rinsing once with PBS and incubated with Enzyme-free Cell Dissociation Buffer (Gibco, Catalog#13151014) for 10 min followed by collection of the cells using a cell scraper and cell viability was assessed using dyes, propidium iodide (PI) or 7-AAD, as detected by fluorescence-activated cell sorting analysis. Shown are the representative histograms of HUVECs treated with Histone 3 only (H3) and HUVECs treated with Histone 3 and APC (H3 + APC) with cell count as y-axis vs. 7-AAD staining intensity as x-axis.

RESTRICTED

Supplementary Table 1. Kinetic constants of anti-APC mAbs (Type I mAb C25K23 and Type II mAb HAPC1573) binding to APC or PPACK-APC by SPR analyses

mAb	Analyte	ka ($M^{-1} sec^{-1}$)	kd (sec^{-1})	K_A (M^{-1})	K_D (M)	Chi^2
Type I	APC	2.12 $x10^5$	1.48 $x10^{-3}$	1.43 $x10^8$	6.98 $x10^{-9}$	1.56
Type I	PPACK-APC	No measurable binding				
Type II	APC	5.78 $x10^5$	4.56 $x10^{-3}$	1.27 $x10^8$	7.89 $x10^{-9}$	2.3
Type II	PPACK-APC	7.47 $x10^5$	4.46 $x10^{-3}$	1.67 $x10^8$	5.97 $x10^{-9}$	0.92

Note: Anti-APC mAbs (C25K23 or HAPC1573) were captured on the chip via immobilized anti-human Fc or anti-mouse Fc capture antibodies, respectively. APC or PPACK-APC were sequentially injected onto the surface as described in the Methods. Binding of APC or PPACK-APC complex were measured at 7 concentrations (20-0.312 nM). Data were fit to a Langmuir 1:1 binding model. Binding kinetic constants: association rate constant (ka), dissociation rate constant (kd), equilibrium association constant (K_A), and equilibrium dissociation constant (K_D) were generated. The chi squared value indicates the fit to the model.

RESTRICTED

Supplementary Table 2. Pharmacokinetic (PK) parameters of anti-APC mAbs in normal cynomolgus monkeys. Type I mAb (BAY1316786) and Type II mAb (BAY1896502) were administered intravenously as a single dose (N=3 animals per dose group). PK parameters are expressed as average values from 3 monkeys ± SD. PK parameters were calculated using Phoenix WinNonlin (Version 6.4, Pharsight, Certara,L.P. Princeton, NJ, USA).

**PK Parameters**	**Type 1 mAb** BAY1316786	**Type II mAb** BAY1896502
**IV Dose (mg/kg)**	3	3
**AUCnorm (Kg*h/L)**	982 ± 272	7121 ± 2166
**CL (ml/h/kg)**	1.1 ± 0.27	0.15 ± 0.047
**VSS (ml/kg)**	120 ± 4.9	55 ± 21
**T1/2 (h)**	111 ± 18	289 ± 158
**MRT (h)**	116 ± 28	399 ± 203

Note: IV Dose, intravenous dose; AUCnorm, dose-normalized area under the curve; CL, clearance; VSS, volume of distribution at steady state; T1/2, half-life; MRT, mean residence time.

RESTRICTED

Supplementary Table 3. Amino acid sequences of monoclonal antibodies (mAbs) and Fabs with annotations (complementarity determining regions in **bold**; constant domain in grey highlight)

Name	Type	Target gene	Chain	Isotype	AA sequence
TPP-2312 BAY1316786	Type I mAb (IgG)	APC	LC	hu lambda	QSVLTQPPSV SGAPGQRVTI SC**TGSSSNIG AAYDVH**WYQQ LPGTAPKLLI Y**GNN KRPS**GV PDRFSGSKSG TSASLAITGL QAEDEADYYC **QSYDSSLSGS V**FGGGTKL TV LGQPKAAPSV TLFPPSSEEL QANKATLVCL ISDFYPGAVT VAWKADSSPV K AGVETTTPS KQSNNKYAAS SYLSLTPEQW KSHRSYSCQV THEGSTVEKT VAPT ECS
			HC	huIgG2	EVQ LLESGGGLVQ PGGSLRLSCA AS**GFTFSSYW MS**WVRQAPGK GLEWVS**GVS W NGARTHYADS VKG**RFTISRD NSKNTLYLQM NSLRAEDTAV YYCAL**TGRSG WMRFPNWFDP** WGQGTLVTVS SASTKGPSVF PLAPCSRSTS ESTAALGCLV KD YFPEPVTV SWNSGALTSG VHTFPAVLQS SGLYSLSSVV TVPSSNFGTQ TYTCNV DHKP SNTKVDKTVE RKCCVECPPC PAPPVAGPSV FLFPPKPKDT LMISRTPEVT CVVVDVSHED PEVQFNWYVD GVEVHNAKTK PREEQFNSTF RVVSVLTVVH QD WLNGKEYK CKVSNKGLPA PIEKTISKTK GQPREPQVYT LPPSREEMTK NQVSLT CLVK GFYPSDIAVE WESNGQPENN YKTTPPMLDS DGSFFLYSKL TVDKSRWQQ G NVFSCSVMHE ALHNHYTQKS LSLSPG
TPP-4885 BAY1896502	Type II mAb (IgG)	APC	LC	hu kappa	DIVLTQS PASLAVSPGQ RATITC**KASE SVDSFGATFM H**WYQQKPGQP PKLLIY**LASN LES**GVPARFS GSGSGTDFTL TINPVEADDT ANYYC**QQNNE DPYT**FGQGTK LEIKRTVAAP SVFIFPPSDE QLKSGTASVV CLLNNFYPRE AKVQWKVDNA LQSGNSQESV TEQDSKDSTY SLSSTLTLSK ADYEKHKVYA CEVTHQGLSS PVTKSFNRGE C
			HC	huIgG2	EVQLVESGGG LVQPGGSLRL SCAAS**GFTFS NYYLN**WVRQA PGKGLEWVG**D IRLKSNNYEK HYAESVKG**RF TISRDDSKNS LYLQMNSLKT EDTAVYYCAR **EGDYFDY**WGQ GTLVTVSSAS TKGPSVFPLA PCSRSTSEST AALGCLVKDY FPEPVTVSWN SGALTSGVHT FPAVLQSSGL YSLSSVVTVP SSNFGTQTYT CNVDHKPSNT KVDKTVERKC CVECPPCPAP PVAGPSVFLF PPKPKDTLMI SRTPEVTCVV VDVSHEDPEV QFNWYVDGVE VHNAKTKPRE EQFNSTFRVV SVLTVVHQDW LNGKEYKCKV SNKGLPAPIE KTISKTKGQP REPQVYTLPP SREEMTKNQV SLTCLVKGFY PSDIAVEWES NGQPENNYKT TPPMLDSDGS FFLYSKLTVD KSRWQQGNVF SCSVMHEALH NHYTQKSLSL SPG
TPP-2781 (C25K23Fab)	Type I Fab	APC	LC	hu lambda	QSVLTQPPSA SGTPGQRVTI SC**TGSSSNIG AAYDVH**WYQQ LPGTAPKLLI Y**GNNKRPS**GV PDRFSGSKSG TSASLAISGL RSEDEADYYC **QSYDSSLSGS V**FGGGTKLTV LGQPKAAPSV TLFPPSSEEL QANKATLVCL ISDFYPGAVT VAWKADSSPV KAGVETTTPS KQSNNKYAAS SYLSLTPEQW KSHRSYSCQV THEGSTVEKT VAPTECS

RESTRICTED

Table cont.					
**Name**	**Type**	**Target gene**	**Chain**	**Isotype**	**AA sequence**
TPP-2781 (C25K23Fab)	Type I Fab	APC	HC	huIgG1	EVQ LLESGGGLVQ PGGSLRLSCA AS**GFTFSSYW MS**WVRQAPGK GLEWVS**GVSW NGSRTHYADS VKG**RFTISRD NSKNTLYLQM NSLRAEDTAV YYCAL**TGRSG WMRFPNWFDP** WGQGTLVTVT SASTKGPSVF PLAPSSKSTS GGTAALGCLV KDYFPEPVTV SWNSGALTSG VHTFPAVLQS SGLYSLSSVV TVPSSSLGTQ TYICNVNHKP SNTKVDKKVE PKSCAAGSEQ KLISEEDLSG SAAAHHHHHH
TPP-5040 (hu1573Fab)	Type II Fab	APC	LC	hu kappa	DIVLTQSPA SLAVSPGQRA TITC**KASESV DSFGATFMH**W YQQKPGQPPK LLIY**LASNLE** SGVPARFSGS GSGTDFTLTI NPVEADDTAN YYC**QQNNEDP YT**FGQGTKLE IKRTVAAPSV FIFPPSDEQL KSGTASVVCL LNNFYPREAK VQWKVDNALQ SGNSQESVTE QDSKDSTYSL SSTLTLSKAD YEKHKVYACE VTHQGLSSPV TKSFNRGEC
			HC	huIgG1	EVQLVESGGG LVQPGGSLRL SCAAS**GFTFS NYYLN**WVRQA PGKGLEWVG**D IRLKSNNYEK HYAESVKG**RF TISRDDSKNS LYLQMNSLKT EDTAVYYCAR **EGDYFDY**WGQ GTLVTVSSAS TKGPSVFPLA PSSKSTSGGT AALGCLVKDY FPEPVTVSWN SGALTSGVHT FPAVLQSSGL YSLSSVVTVP SSSLGTQTYI CNVNHKPSNT KVDKKVEPKS C
TPP-5958 (BO2C11-cynoIgG1)	IgG	FVIII C2 domain	LC	cyno kappa	E IALTQSPGTL SLSPGERATL SC**RASQSFSS SYLA**WYQQKP GQAPRLLIY**G AST RAT**GIPD RFSGSGSGTD FTLTISRLEP EDFAVYYC**QK YGTSAIT**FGQ GTRLEIKR AV AAPSVFIFPP SEDQVKSGTV SVVCLLNNFY PREASVKWKV DGALKTGNSQ E SVTEQDSKD NTYSLSSTLT LSSTDYQSHN VYACEVTHQG LSSPVTKSFN RGEC
			HC	cyno IgG1	QVQLVQSGAE VKKPGASVKV SCKVS**GYTLT ELPVH**WVGQA PGKGLEWVGS **F DPESGESIY AREFQG**SVTM TADTSTDIAY MELSSLRSDD TAVYYCAV**PD PDAF DI**WGQG TMVTVSSAST KGPSVFPLAP SSRSTSESTA ALGCLVKDYF PEPVTVSW NS GSLTSGVHTF PAVLQSSGLY SLSSVVTVPS SSLGTQTYVC NVNHKPSNTK VD KRVEIKTC GGGSKPPTCP PCPAPELLGG PSVFLFPPKP KDTLMISRTP EVTCVVV DVS QEDPDVKFNW YVNGAEVHHA QTKPRETQYN STYRVVSVLT VTHQDWLN GK EYTCKVSNKA LPAPIQKTIS KDKGQPREPQ VYTLPPSREE LTKNQVSLTC LV KGFYPSDI VVEWESSGQP ENTYKTTPPV LDSDGSYFLY SKLTVDKSRW QQGNV FSCSV MHEALHNHYT QKSLSVSPG

RESTRICTED
