# Supplementary material for: Targeted inhibition of activated protein C by a non-active-site inhibitory antibody to treat hemophilia
Source: Nat Commun. 2020 Jun 12;11:2992. doi: 10.1038/s41467-020-16720-9 (PMC7293249; doi:10.1038/s41467-020-16720-9)
Supplement: Supplementary file 2 — Reporting Summary [file 41467_2020_16720_MOESM2_ESM.pdf]

nature research

Corresponding author(s): NCOMMS-19-1755118-B Xiao-Yan Zhao, Laurent O. Mosnier and Volker Laux

Last updated by author(s): May 11, 2020

# Reporting Summary

Nature Research wishes to improve the reproducibility of the work that we publish. This form provides structure for consistency and transparency in reporting. For further information on Nature Research policies, see Authors & Referees and the Editorial Policy Checklist .

## Statistics

For all statistical analyses, confirm that the following items are present in the figure legend, table legend, main text, or Methods section.

| n/a | Confirmed | |
|---|---|---|
| ☐ | ☒ | The exact sample size (*n*) for each experimental group/condition, given as a discrete number and unit of measurement |
| ☐ | ☒ | A statement on whether measurements were taken from distinct samples or whether the same sample was measured repeatedly |
| ☐ | ☒ | The statistical test(s) used AND whether they are one- or two-sided<br>*Only common tests should be described solely by name; describe more complex techniques in the Methods section.* |
| ☒ | ☐ | A description of all covariates tested |
| ☒ | ☐ | A description of any assumptions or corrections, such as tests of normality and adjustment for multiple comparisons |
| ☒ | ☐ | A full description of the statistical parameters including central tendency (e.g. means) or other basic estimates (e.g. regression coefficient) AND variation (e.g. standard deviation) or associated estimates of uncertainty (e.g. confidence intervals) |
| ☒ | ☐ | For null hypothesis testing, the test statistic (e.g. *F*, *t*, *r*) with confidence intervals, effect sizes, degrees of freedom and *P* value noted<br>*Give P values as exact values whenever suitable.* |
| ☒ | ☐ | For Bayesian analysis, information on the choice of priors and Markov chain Monte Carlo settings |
| ☒ | ☐ | For hierarchical and complex designs, identification of the appropriate level for tests and full reporting of outcomes |
| ☒ | ☐ | Estimates of effect sizes (e.g. Cohen's *d*, Pearson's *r*), indicating how they were calculated |

*Our web collection on statistics for biologists contains articles on many of the points above.*

## Software and code

Policy information about availability of computer code

| | |
|---|---|
| Data collection | ELISA: Softmax Pro 5.2; FACS: Flow Jo (TreeStar); X-ray structure: Blu-Ice (5.0); iCelligence: RTCA 2.0 (Acea); Odyssey: Image Studio v5.2 (LiCor). |
| Data analysis | ELISA: Softmax Pro 5.2; SPR: Biacore T200 Evaluation Software version 3.0; in vitro data: Excel and GraphPad Prism, in vivo: GraphPad Prism; X-ray structure: HKL2000 (HKLSuite0.95SITE); CCP4(CCP4Interface 2.2.0); COOT (0.8.2); PHENIX (phenix.refine: 1.9_1692); PyMol (1.8.0.0); iCelligence: RTCA 2.0 (Acea); Odyssey: Image Studio v5.2 (LiCor). |

For manuscripts utilizing custom algorithms or software that are central to the research but not yet described in published literature, software must be made available to editors/reviewers. We strongly encourage code deposition in a community repository (e.g. GitHub). See the Nature Research guidelines for submitting code & software for further information.

## Data

Policy information about availability of data

All manuscripts must include a data availability statement. This statement should provide the following information, where applicable:

- Accession codes, unique identifiers, or web links for publicly available datasets
- A list of figures that have associated raw data
- A description of any restrictions on data availability

The data that support the findings of this study are available from the corresponding authors upon reasonable request. Any Supplementary Information and Source Data files are available in the online version of the paper. The atomic coordinates and structure factors of the hAPC-C25K23 Fab and hAPC-h1573 Fab complex structures have been deposited in the Protein Data Bank (PDB) under accession codes 6M3B and 6M3C.

# Field-specific reporting

Please select the one below that is the best fit for your research. If you are not sure, read the appropriate sections before making your selection.

- [x] Life sciences
- [ ] Behavioural & social sciences
- [ ] Ecological, evolutionary & environmental sciences

For a reference copy of the document with all sections, see nature.com/documents/nr-reporting-summary-flat.pdf

# Life sciences study design

All studies must disclose on these points even when the disclosure is negative.

| | |
|---|---|
| Sample size | In vitro assays were established as part of a pre-clinical screening program used to characterize multiple discovery antibodies as part of a drug development project. These well-established assays were used to characterize the antibodies found in this publication. N=2-3 were used as part of the screening process with duplicate analysis of a sample as standard part of aPTT and TGA procedures for each N, including the data presented here. Since these assays were part of a screening cascade with multiple antibodies tested and corresponding data in multiple assays, the limited N was considered sufficient.<br><br>The N selected for the in vivo studies was based on pilot NHP studies with tool compounds and the number of animals needed to reach a statistically significant outcome. The pilot data with tool compounds and reproduction of those findings in the studies presented here supported the N selection for the in vivo studies. |
| Data exclusions | No data were excluded for analysis. |
| Replication | All attempts at replication were successful. |
| Randomization | Cynomolgus monkeys were randomized by evenly distributing the weights per group. |
| Blinding | The dosing solutions were made by people outside the vivarium and then handed to the animal technicians inside, blinded.  So the techs supposedly did not know what they were dosing. |

# Reporting for specific materials, systems and methods

We require information from authors about some types of materials, experimental systems and methods used in many studies. Here, indicate whether each material, system or method listed is relevant to your study. If you are not sure if a list item applies to your research, read the appropriate section before selecting a response.

## Materials & experimental systems

| n/a | Involved in the study | |
|---|---|---|
| [ ] | [x] | Antibodies |
| [ ] | [x] | Eukaryotic cell lines |
| [x] | [ ] | Palaeontology |
| [ ] | [x] | Animals and other organisms |
| [x] | [ ] | Human research participants |
| [x] | [ ] | Clinical data |

## Methods

| n/a | Involved in the study | |
|---|---|---|
| [x] | [ ] | ChIP-seq |
| [ ] | [x] | Flow cytometry |
| [x] | [ ] | MRI-based neuroimaging |

## Antibodies

| | |
|---|---|
| Antibodies used | The mouse anti-hAPC antibody HAPC1573 from Oklahoma Medical Research Foundation was humanized to generate the antiAPC type II mAb. The type I mAb was identified by panning the n-CoDeR® phage-display library of human antibody Fab fragment purchased from BioInvent International AB, Sweden. |
| Validation | Antibodies were validated by antigen-antibody binding assays (ELISA, SPR) and DNA sequencing, as well as by APC chromogenic assay/FVa inactivation assay/aPTT clotting assay/TGA. |

## Eukaryotic cell lines

Policy information about cell lines

| | |
|---|---|
| Cell line source(s) | HAPC1573 hybridoma cell line from Charles Esmon (Oklahoma Medical Research Foundation, Oklahoma City, OK, USA); QMCF (CHO-based cell line) from Icosagen (San Francisco, CA, USA); HUVEC and EA.hy.926 cell lines from ATCC. |

| | |
|---|---|
| Authentication | Hybridoma cell line: the HAPC1573 purified from this cell line was extensively characterized and behaved as expected. The commercial QMCF, HUVEC, and EA.hy.926 cell lines were authenticated by morphology and growth characteristics. |
| Mycoplasma contamination | All cell lines were tested negative for mycoplasma contamination. |
| Commonly misidentified lines (See ICLAC register) | No commonly misidentified cell lines were used. |

## Animals and other organisms

Policy information about studies involving animals; ARRIVE guidelines recommended for reporting animal research

| | |
|---|---|
| Laboratory animals | Cynomolgus monkeys, approx 3 years old males |
| Wild animals | Did not involve wild animals |
| Field-collected samples | Did not involve samples collected from the field |
| Ethics oversight | All study protocols were approved by the Institutional Animal Care and Use Committee and were conducted in accordance with the Guide for the Care and Use of Laboratory Animals |

Note that full information on the approval of the study protocol must also be provided in the manuscript.

## Flow Cytometry

### Plots

Confirm that:

- [x] The axis labels state the marker and fluorochrome used (e.g. CD4-FITC).
- [x] The axis scales are clearly visible. Include numbers along axes only for bottom left plot of group (a 'group' is an analysis of identical markers).
- [x] All plots are contour plots with outliers or pseudocolor plots.
- [x] A numerical value for number of cells or percentage (with statistics) is provided.

### Methodology

| | |
|---|---|
| Sample preparation | HVUEC cells (ATCC) were rinsed and incubated with Cell Dissociation Buffer (GIBCO, enzyme free) for 5 min, followed by manual harvesting using cell scraper. Cell viability was assessed using dyes, PI or 7-AAD (BD Bioscience). |
| Instrument | BD LSRII- 3 laser system (405, 488, 640 nm) |
| Software | Flow Jo (TreeStar) |
| Cell population abundance | Assay used single cell type |
| Gating strategy | 1. FCS/SCS adjusted to identify single cell (R1) 2. Dead cells were permeable to 7-AAD dye and stained positive inFL3 channel (546/647; Ex/Em) |

- [x] Tick this box to confirm that a figure exemplifying the gating strategy is provided in the Supplementary Information.
